# Changes in REVEAL risk score in patients with pulmonary arterial hypertension treated with macitentan in clinical practice: results from the PRACMA study

**DOI:** 10.1186/s12890-020-01197-5

**Published:** 2020-06-02

**Authors:** Pilar Escribano-Subias, Raquel López, Luis Almenar, María Lázaro, Ian Forn, Anna Torrent, Isabel Blanco, Joan Albert Barberà, on behalf of the PRACMA investigators, Luis Almenar, José Manuel Alvarez Dobaño, Adolfo Baloira, Julia Barbado, Pedro Bedate Diaz, Isabel Blanco, David Blanquer, Ana José Bustamante Ruiz, Sergio Cadenas, Ignacio Casado, Carlos Chamorro, José Manuel Cifrian, David Cremer, Eva Delgado, Juan Luis Delgado, Juan Antonio Domingo, Pilar Escribano, Luis García Arangüena, Juan Pablo García Muñoz, David Iturbe, Antonio Lara, María Lázaro, Manuel López Meseguer, Raquel López, Ana Madroñero, Adela Marín, Lluis Molina Ferragut, Eduardo Moreno Escobar, Ana Núñez, Juan Ortiz de Saracho y Bobo, Cilia Amparo Peralta, Gregorio Pérez Peñate, Javier Pomares Amigó, Carlos Rodriguez, Vicente Roig, Ximo Rueda, Ernest Sala, Rafaela Sánchez Simón

**Affiliations:** 1 Pulmonary Hypertension Unit, Cardiology Department, Hospital Universitario 12 de Octubre, Av. Córdoba, s/n, 28041 Madrid, Spain; 2 CIBER de Enfermedades Cardiovasculares (CIBERCV), Instituto de Investigación Sanitaria Hospital 12 de Octubre (imas12), Av. Córdoba, s/n, 28041 Madrid, Spain; 3 Pulmonary Service, Hospital Universitari i Politècnic La Fe, València, Spain; 4 Cardiology Department, Hospital Virgen de la Salud, Toledo, Spain; 5 Actelion Pharmaceuticals España S.L., Barcelona, Spain; 6 Pulmonary Service, Hospital Clínic, Institut d’Investigacions Biomèdiques August Pi i Sunyer (IDIBAPS), Barcelona, Spain

**Keywords:** Macitentan, Observational study, Pulmonary arterial hypertension, Risk assessment

## Abstract

**Background:**

Macitentan is a dual endothelin receptor antagonist indicated for the long-term treatment of pulmonary arterial hypertension (PAH). We evaluated the change over time in REVEAL risk score in incident and prevalent patients receiving macitentan for the first time.

**Methods:**

Retrospective, observational study including adult patients with idiopathic/heritable PAH or PAH associated with connective tissue disorders or congenital heart disease treated with macitentan for ≥6-month follow-up in Spain. The REVEAL risk score and risk strata were computed at the start of macitentan and after ≥6-month in patients with ≥7 out of 12 valid REVEAL components.

**Results:**

Overall, 81 patients (57 for the REVEAL score) were analysed, 77.8% women. The mean age was 57.2 years and 50.6% of patients had idiopathic/heritable PAH. Prevalent patients were 59.3 and 40.7% were incident. Main therapies for PAH included macitentan monotherapy (42.0%) and macitentan in combination with phosphodiesterase type 5 inhibitor (44.4%). With a median time of macitentan treatment of 10.5 months, the mean REVEAL score was 8.7 points at baseline and was 7.2 points after ≥6-month follow-up. The mean change (95% CI) in REVEAL risk score was − 1.4 (− 2.0, − 0.9) points (*p* < 0.0001), being − 1.8 (− 3.0, − 0.7) points (*p* = 0.0040) and − 1.2 (− 1.8, − 0.5) points (*p* = 0.0010), in incident and prevalent patients, respectively. The reduction was also significant by risk stratum (36.8% of patients in the high-very high risk strata at baseline versus 14.0% after ≥6-month, *p* < 0.05) and therapy group. The REVEAL components that significantly improved were WHO functional class (FC) (63.9% FC III at macitentan initiation and 23.6% after ≥6-month, p < 0.0001), 6-min walk test (mean change: 41.8 m, *p* < 0.01), brain natriuretic peptide (BNP) or N-terminal proBNP (NT-proBNP) (mean change of − 157.6 pg/mL and − 530.0 pg/mL, respectively, p < 0.05 both), and pulmonary vascular resistance (PVR) (mean change: − 3.4 WU, p < 0.01).

**Conclusions:**

In this study, treatment with macitentan improved the REVEAL risk strata and score in both incident and prevalent PAH patients, and in all patients regardless of the therapy strategy. Macitentan significantly improved some of REVEAL components including WHO FC, BNP/NT-proBNP, PVR, and 6-min walk test after at least 6-month follow-up.

## Background

Pulmonary arterial hypertension (PAH) is a progressive disease characterised by an increased mean pulmonary arterial pressure of at least 25 mmHg at rest and a pulmonary vascular resistance (PVR) greater than 3 Wood units (WU) [1, 2] with low incidence and prevalence in the general population [3, 4], and a poor prognosis without therapy in terms of mortality [2]. In Spain, the estimated incidence of PAH ranges from 2.4 to 7.6 cases/million/year [5]. The 1-year survival rate of newly diagnosed PAH patients may be predicted using a risk score calculator derived from the Registry to Evaluate Early and Long-term PAH Disease Management (REVEAL) [6, 7]. Data from this registry reported that PAH patients had 1-year survival rate of 85% and 5-year survival rate of 57% from the time of diagnosis [8].

Endothelin receptor antagonists emerged as an important therapeutic option for PAH in the late 1990s [9]. Among these agents, macitentan is an oral agent with dual endothelin receptor antagonist function, which is indicated as monotherapy or in combination for the long-term treatment of PAH in adult patients of World Health Organization (WHO) functional class (FC) II to III. Efficacy has been shown in a PAH population including idiopathic and heritable PAH, PAH associated with connective tissue diseases, and PAH associated with repaired congenital heart disease [10].

As REVEAL risk score changes over time in most PAH patients, ongoing risk score assessments can identify changes in disease course and thus, help optimizing therapeutic strategies in PAH [11]. The primary objective of this observational study was to assess the change over a minimum period of six months in REVEAL risk score and risk strata in PAH patients receiving macitentan for the first time in Spain. The secondary objectives were to describe patients’ characteristics and treatment patterns, and assess the changes in individual parameters of the REVEAL score components in PAH patients.

## Methods

The PRACMA was a retrospective observational study with at least 6-month follow-up. The inclusion criteria were adult patients with idiopathic or heritable PAH, PAH associated with connective tissue disorders or PAH associated with corrected simple congenital heart disease of WHO FC II to III, and treated for the first time with macitentan 10 mg once daily, either in monotherapy or in combination, for at least six months. The protocol was approved by the independent ethics committee at each study site.

The study was conducted in 28 centres from Spain between September 2016 and September 2017. Study data was collected from medical records, with data collected at baseline (within a maximum of 4-month prior to the start of macitentan) and at least 6-month (175 days) after treatment initiation. Demographics (gender, age), WHO group I subgroup (idiopathic or heritable PAH, PAH associated with connective tissue disorders, or PAH associated with corrected simple congenital heart disease), PAH diagnosis date, onset date for macitentan as well as other therapy for PAH were collected at baseline. The following variables were collected at both baseline and ≥ 6-month time points: presence of renal insufficiency, WHO FC (I to IV), vital signs (systolic blood pressure [SBP] and heart rate), 6-min walk test, laboratory data (brain natriuretic peptide [BNP] or N-terminal proBNP [NT-proBNP]), echocardiogram (pericardial effusion), pulmonary function test data (diffusing capacity of the lungs for carbon monoxide [DLCO]), and hemodynamic parameters related to right-heart catheterization (mean right atrial pressure [mRAP] and PVR).

### Statistical analysis

The evaluable population comprised those patients with at least 175 days of treatment with macitentan and at least one valid value for any of the components of the REVEAL score (WHO FC, vital signs, 6-min walk test, laboratory data, echocardiogram, pulmonary function test, or hemodynamic parameters) either at baseline or at ≥6-month time points.

Due to the inclusion of similar ratio of incident and prevalent patients, it was decided to analyse them separately. Incident patients were defined as patients diagnosed less than six months prior to the start date of macitentan. Patients were defined as prevalent if the PAH diagnosis date was equal or greater than six months prior to the start date of macitentan.

Descriptive analyses were provided for all variables. The presence of renal insufficiency was based on the comorbidity being recorded in the patient’s medical record. The REVEAL risk score was computed at baseline and at ≥6-month time points according to the REVEAL risk score calculator and selecting patients with at least 7 out of 12 valid REVEAL components [6] at both time points (REVEAL population). All men who turned > 60 years old during the course of the ≥6-month period were counted in the “Men aged >60 years” category. No missing imputation was performed. Risk scores ranged from 0 (lowest risk) to 22 (highest risk). Risk categories by risk score were 1–7 low risk, 8 average risk, 9 moderate high risk, 10 or 11 high risk, and ≥ 12 very high risk.

For analysis of risk categories, the average and moderate high categories were grouped [8, 9] as well as the high and very high categories (≥10) due to the low frequencies in these categories. REVEAL score was analysed computing the mean change between ≥6-month and baseline REVEAL score and corresponding 95% confidence interval. For continuous components the change between visits has been addressed computing a point estimate of mean and median difference between baseline and ≥ 6-month as well as their confidence interval. For categorical components we have computed the proportion of events at both baseline and ≥ 6-month visits and corresponding 95% confidence interval for the difference. Wilcoxon matched pairs signed rank test and t-paired test were applied and a sensitivity analysis excluding three outliers for change was performed.

For the comparisons between incident and prevalent patients, Mann–Whitney U test and chi-square test were used for continuous and categorical variables, respectively. For continuous individual REVEAL components, changes over time were compared with the Wilcoxon matched-pairs signed-rank test. For categorical REVEAL components, changes over time were compared with the McNemar test. A *p*-value of < 0.05 was considered significant.

Statistical analyses were performed with the Stata Statistical Software version 15 (College Station, TX: StataCorp LLC).

## Results

### Baseline characteristics

**Fig. 1:**
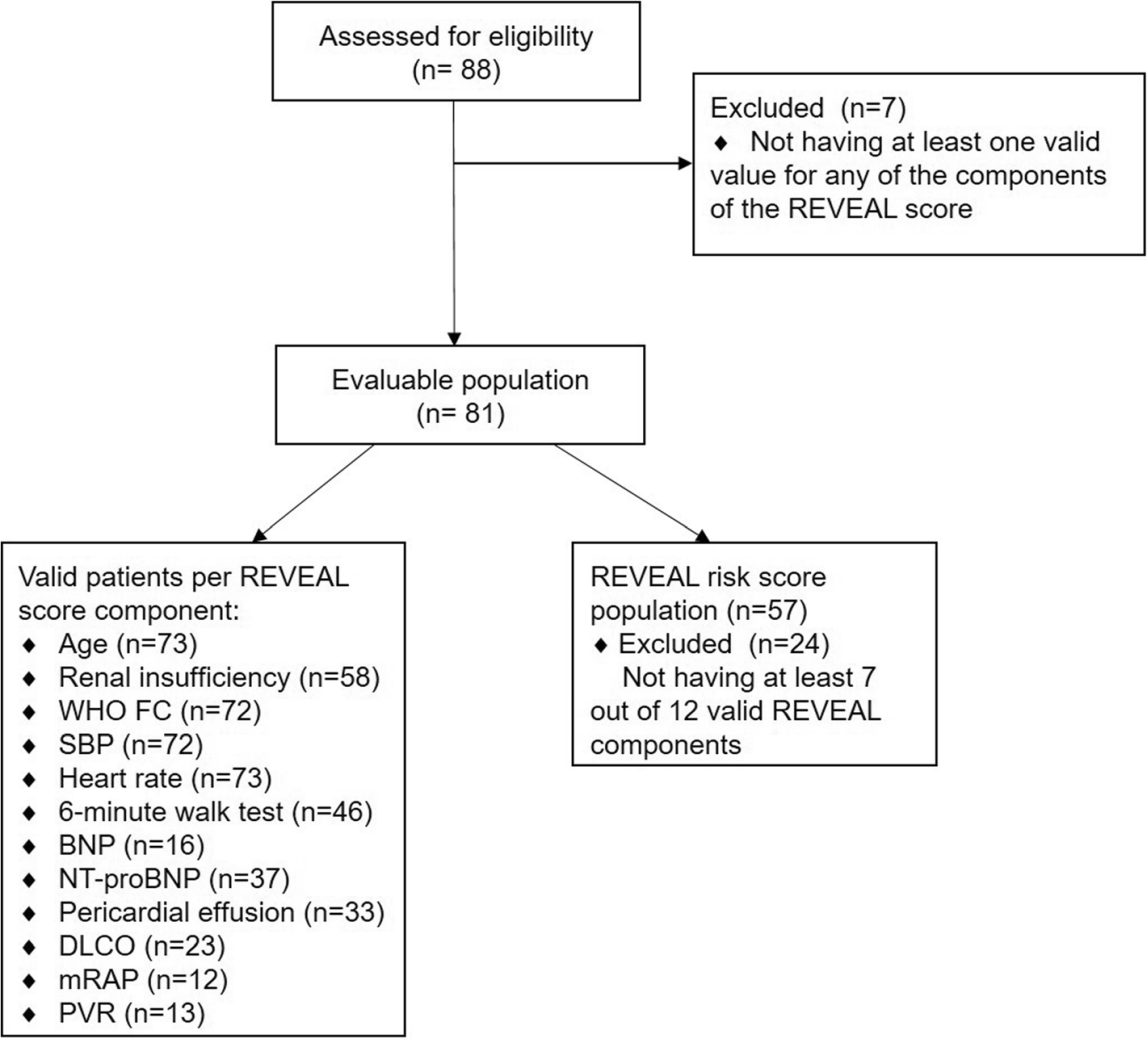
Patient flow. BNP = brain natriuretic peptide; DLCO = diffusing capacity of the lungs for carbon monoxide; FC = functional class; mRAP = mean right atrial pressure; NT-proBNP=N-terminal pro-BNP; PVR = pulmonary vascular resistance; SBP = systolic blood pressure; WHO=World Health Organization

A total of 88 patients were enrolled and 81 were included in the analysis (mean of 2.9 patients per site), of whom 57 (70.4% of evaluable population) were valid to compute the REVEAL risk score (Fig. 1).

Table 1 shows the main characteristics of the study cohorts at baseline. Prevalent patients were 59.3% of the overall population. The population was mostly female (77.8%), and mean age of the overall population was 57.2 years. Idiopathic or heritable PAH was the most common PAH subgroup with 50.6% of patients. Forty-two percent of patients were on macitentan as monotherapy (66.7% of incident patients and 25.0% of prevalent patients) and 44.4% were on combination with a phosphodiesterase type 5 (PDE5) inhibitor (33.3% of incident patients and 52.1% of prevalent patients), mainly sildenafil (80.6%). Baseline characteristics were comparable between groups except that prevalent patients had significantly greater distance covered in the 6-min walk test than incident patients (*p* < 0.01).

**Table 1:** Patient demographics and disease characteristics at baseline

	Incident (*n* = 33)	Prevalent (*n* = 48)	Total (*n* = 81)
Age, years, mean (SD)	57.9 (18.3)	56.6 (14.9)	57.2 (16.3)
Sex, female	24 (72.7)	39 (81.3)	63 (77.8)
WHO group I subgroup			
Idiopathic or heritable PAH	17 (51.5)	24 (50.0)	41 (50.6)
PAH associated with connective tissue disorders	15 (45.5)	14 (29.2)	29 (35.8)
PAH associated with corrected simple congenital heart disease	1 (3.0)	9 (18.8)	10 (12.4)
Time since PAH diagnosis, months, median (Q1-Q3)^*^	1.0 (0.2–2.6)	42.8 (14.5–97.7)	11.1 (1.8–56.2)
WHO FC			
I	1 (3.0)	2 (4.2)	3 (3.7)
II	5 (15.1)	13 (27.1)	18 (22.2)
III	21 (63.6)	30 (62.5)	51 (63.0)
IV	6 (18.2)	3 (6.3)	9 (11.1)
Renal insufficiency, yes	4 (13.3)	9 (19.6)	13 (17.1)
SBP, mm Hg, mean (SD)	117.9 (16.0)	122.2 (17.9)	120.4 (17.2)
Heart rate, beats/min, mean (SD)	79.5 (11.8)	77.9 (12.6)	78.6 (12.3)
6-min walk test, m, mean (SD)^*^	309.1 (153.2)	401.6 (102.9)	362.5 (133.7)
BNP, pg/mL, mean (SD)	368.7 (433.2)	247.5 (188.5)	300.2 (316.1)
NT-proBNP, pg/mL mean (SD)	1140.9 (1451.3)	1452.4 (1995.5)	1327.9 (1797.6)
Pericardial effusion, yes	4 (12.9)	4 (8.9)	8 (10.5)
DLCO, % predicted, mean (SD)	57.4 (19.3)	57.9 (19.4)	57.8 (19.2)
mRAP, mm Hg, mean (SD)	11.7 (10.7)	10.3 (8.1)	10.9 (9.3)
PVR, WU, mean (SD)	9.2 (4.5)	8.8 (4.3)	9.0 (4.3)
Therapy for PAH^*^			
Macitentan monotherapy	22 (66.7)	12 (25.0)	34 (42.0)
Macitentan + PDE5 inhibitor	11 (33.3)	25 (52.1)	36 (44.4)
Macitentan + prostacyclin / Macitentan + PDE5 inhibitor + prostacyclin	0 (0)	11 (22.9)	11 (13.6)

Data are number of patients (percentage) except when otherwise indicated

*BNP* brain natriuretic peptide, *DLCO* diffusing capacity of the lungs for carbon monoxide, *FC* functional class, *mRAP* mean right atrial pressure, *NT-proBNP* N-terminal pro-BNP, *PAH* pulmonary arterial hypertension, *PDE5* phosphodiesterase type 5, *PVR* pulmonary vascular resistance, *Q1* 25th percentile, *Q3* 75th percentile, *SD* standard deviation, *SBP* systolic blood pressure, *WHO* World Health Organization, *WU* Wood units

^*^p < 0.01 between incident and prevalent patients

Therapy for PAH was related to time since diagnosis. Patients on monotherapy (of the patients on monotherapy, 64.7% were incident and 35.3% were prevalent) started macitentan after a median (Q1-Q3) of 3.3 (0.4–13.7) months after PAH diagnosis while patients on combination with a PDE5 inhibitor (of the patients on this combination, 30.6% were incident and 69.4% were prevalent) initiated macitentan after a median (Q1-Q3) of 23.0 (2.6–59.0) months. All patients on double or triple combination therapy with a prostacyclin were prevalent with a median (Q1-Q3) time from PAH diagnosis to start of macitentan of 14.5 (8.8–120.2) months. The median (Q1-Q3) time of macitentan treatment was 10.5 (8.1–13.1) months.

### Changes in REVEAL risk score

At baseline, the mean REVEAL score was 8.7 points, and at ≥6-month time point, it had decreased to 7.2 points. Overall, the mean change (95% confidence interval) in REVEAL risk score over a period of at least six months was − 1.4 (− 2.0, − 0.9) points (*p* < 0.0001), and there was a significant reduction in REVEAL score for incident and prevalent patients (Table 2). According to treatment, the REVEAL risk score reduction was − 1.2 (− 2.0, − 0.5) points (*p* = 0.0050) and − 1.6 (− 2.7, − 0.5) points (*p* = 0.0070) and, in patients on macitentan monotherapy and patients on combination with PDE5 inhibitor, respectively (Table 2).

**Table 2:** Changes in REVEAL risk score at ≥6-month time point: overall and by time since diagnosis and PAH therapy

	Baseline score, mean (SD)	≥6-month score, mean (SD)	Mean change (95% CI)	*p*-value
Total (*n* = 57)	8.7 (2.4)	7.2 (2.4)	−1.4 (−2.0, −0.9)	**< 0.0001**
Macitentan monotherapy (*n* = 25)	7.8 (2.2)	6.5 (2.4)	−1.2 (− 2.0, − 0.5)	**0.0050**
Macitentan + PDE5 inhibitor (*n* = 25)	9.5 (2.3)	7.9 (2.5)	−1.6 (−2.7, − 0.5)	**0.0070**
Macitentan + prostacyclin / Macitentan + PDE5 inhibitor + prostacyclin (*n* = 7)	8.9 (2.3)	7.4 (2.2)	−1.4 (−2.7, −0.1)	**0.0308**
Incident (*n* = 23)	8.9 (2.7)	7.0 (2.3)	−1.8 (−3.0, −0.7)	**0.0040**
Macitentan monotherapy (*n* = 15)	7.9 (2.2)	6.6 (2.2)	−1.3 (−2.5, − 0.2)	**0.0300**
Macitentan + PDE5 inhibitor (*n* = 8)	10.6 (2.7)	7.9 (2.4)	−2.8 (−5.8, − 0.3)	0.0650
Prevalent (*n* = 34)	8.5 (2.2)	7.4 (2.6)	−1.2 (− 1.8, −0.5)	**0.0010**
Macitentan monotherapy (*n* = 10)	7.5 (2.3)	6.4 (2.8)	−1.1 (−2.2, 0.04)	0.0707
Macitentan + PDE5 inhibitor (*n* = 17)	9.0 (2.0)	8.0 (2.6)	−1.1 (−2.1, −0.04)	0.0516
Macitentan + prostacyclin / Macitentan + PDE5 inhibitor + prostacyclin (*n* = 7)	8.9 (2.3)	7.4 (2.2)	−1.4 (−2.7, −0.1)	**0.0308**

Significant *p*-values are marked in bold

*CI* confidence interval, *PDE5* phosphodiesterase type 5, *SD* standard deviation

The REVEAL risk score decreased (improved), remained unchanged, and increased (worsened) in 57.9, 26.3, and 15.8% of patients after at least 6-month follow-up, respectively. The improvement in the REVEAL risk score was similar in both cohorts with 60.9% of incident patients and 55.9% of prevalent patients who improved the risk score after a minimum of 6-month follow-up.

### Changes in REVEAL risk strata

**Fig. 2:**
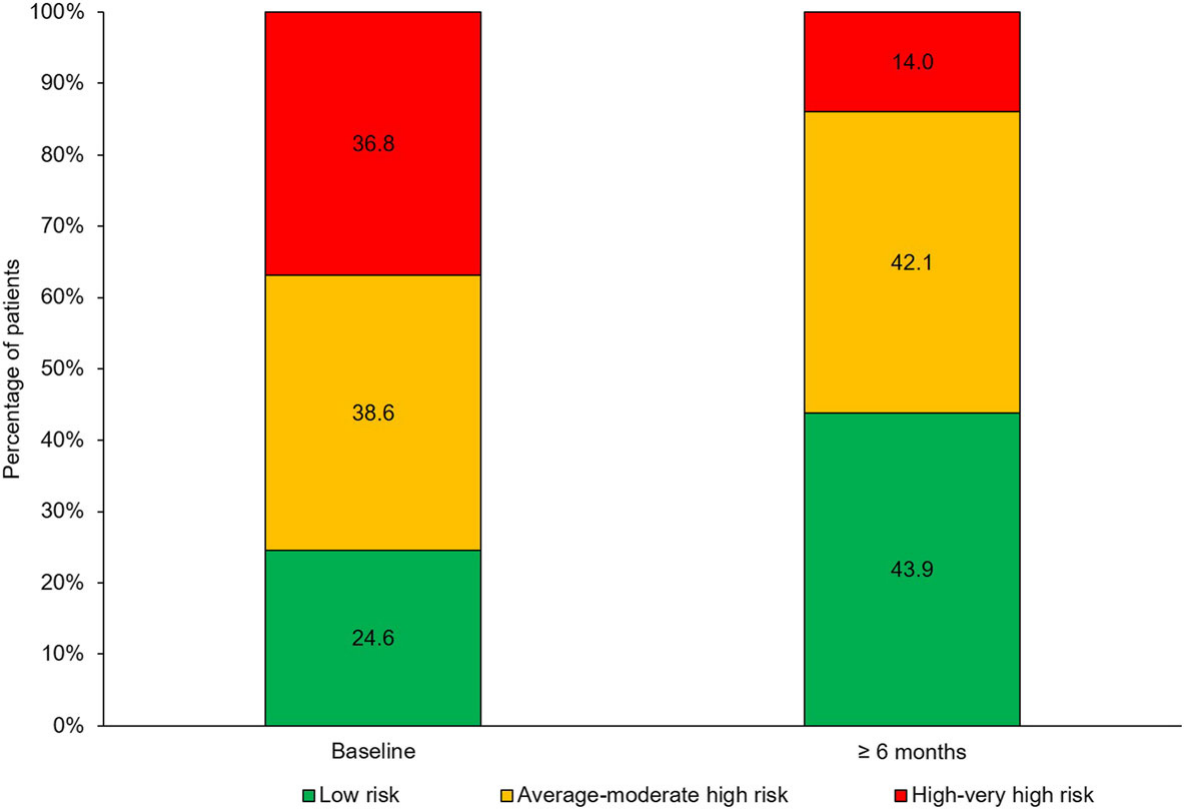
Evolution of REVEAL risk strata after at least six months on macitentan. Grouped risk categories: 1–7 low risk, 8–9 average-moderate high risk, ≥10 high-very high risk

Evolution of REVEAL risk score by risk stratum is shown in Table 3 and Fig. 2. After at least six months from baseline, the number of patients who were in the low and average-moderate high risk strata significantly increased (from 63.2% [*n* = 36] to 86.0% [*n* = 49]) while the number of patients who were in the high-very high risk strata decreased (from 36.8% [*n* = 21] to 14.0% [*n* = 8]) (Fig. 2).

**Table 3:** Distribution of patients by REVEAL risk categories at baseline and ≥ 6-month time points in patients on macitentan

	≥6-month					
Baseline	1–7	8	9	10–11	≥12	Total
1–7	12	2	0	0	0	14
8	5	2	3	0	0	10
9	3	3	6	0	0	12
10–11	4	4	3	5	1	17
≥12	1	0	1	2	0	4
Total	25	11	13	7	1	57

Risk categories: 1–7 low risk, 8 average risk, 9 moderate high risk, 10 or 11 high risk, and ≥ 12 very high risk

Patients with low REVEAL risk were mainly treated with macitentan monotherapy (around 60% of patients both at baseline and after at least 6-month follow up) while patients with high or very high REVEAL risk score were on combination with macitentan and a PDE5 inhibitor (nearby 60% of patients both at baseline and after at least 6-month follow up).

### Individual components of the REVEAL score

**Fig. 3:**
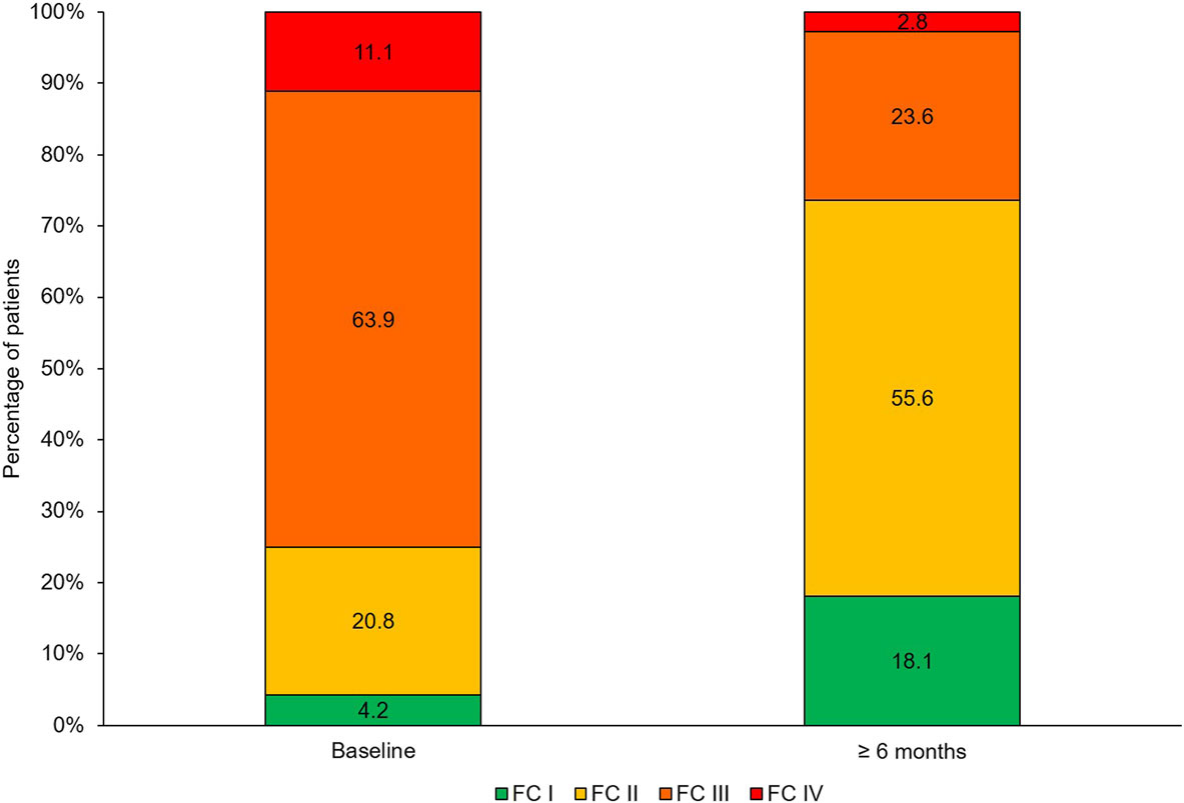
Evolution of WHO functional class (FC) after at least six months on macitentan

**Fig. 4:**
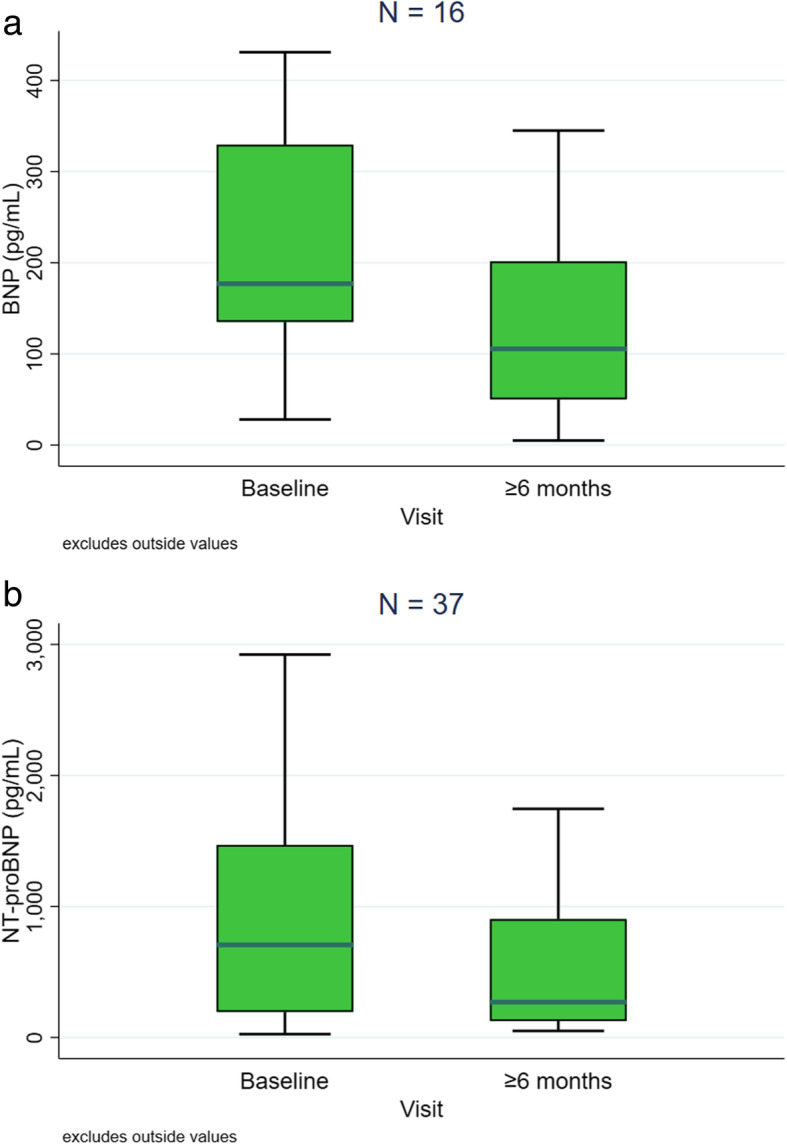
Box plots for brain natriuretic peptide (BNP) [**a**] and N-terminal proBNP (NT-proBNP) [**b**] data at baseline and ≥ 6-month time points in patients on macitentan. The box spans data values between the two quartiles (25th and 75th percentiles), with the horizontal line within the box marking the median value. The upper and lower limits represent 75th percentile + (interquartile range*1.5) and 25th percentile - (interquartile range*1.5), respectively

The main REVEAL individual components that significantly improved after at least six months were (Table 4): WHO FC (63.9% FC III at the start of macitentan versus 23.6% after ≥6-month period, *p* < 0.0001 [Fig. 3]), 6-min walk test (mean change of 41.8 m, *p* < 0.01), BNP (mean change of − 157.6 pg/mL, *p* < 0.05), NT-proBNP (mean change of − 530.0 pg/mL, p < 0.01), and PVR (mean change of − 3.4 WU, p < 0.01). Box plots for BNP and NT-proBNP values are shown in Fig. 4.

**Table 4:** Changes in individual REVEAL components at ≥6-month time point

Component	n	Baseline score, mean (SD)	≥6-month score, mean (SD)	Mean change (95% CI)	*p*-value
WHO FC (as continuous)	72	2.1 (0.7)	2.8 (0.7)	−0.7 (−0.8, −0.5)	**< 0.0001**
SBP, mm Hg	72	120.4 (17.1)	117.9 (15.6)	−2.5 (−6.4, 1.4)	**0.0476**
Heart rate, beats/min	73	77.8 (12.1)	77.0 (13.8)	−0.8 (−4.3, 2.8)	0.5057
6-min walk test, m	46	364.5 (133.3)	406.3 (112.3)	41.8 (14.5, 69.2)	**0.0003**
BNP, pg/mL	16	310.1 (350.2)	152.5 (164.8)	−157.6 (− 336.3, −21.1)	**0.0229**
NT-proBNP, pg/mL	37	1121.1 (1497.4)	590.6 (630.4)	−530.0 (− 972.0, −88.9)	**0.0014**
DLCO, % predicted	23	52.7 (18.0)	50.8 (19.2)	−1.9 (− 1.6, 5.5)	0.2753
mRAP, mm Hg	12	7.9 (4.0)	7.7 (2.6)	−0.3 (−1.6, 2.1)	0.4939
PVR, WU	13	11.5 (4.6)	8.1 (3.4)	−3.4 (−6.5, −0.2)	**0.0033**

Significant *p*-values are marked in bold

*BNP* brain natriuretic peptide, *CI* confidence interval, *DLCO* diffusing capacity of the lungs for carbon monoxide, *FC* functional class, *mRAP* mean right atrial pressure, *NT-proBNP* N-terminal pro-BNP, *PAH* pulmonary arterial hypertension, *PVR* pulmonary vascular resistance, *Q1* 25th percentile, *Q3* 75th percentile, *SD* standard deviation, *SBP* systolic blood pressure, *WHO* World Health Organization, *WU* Wood units

The greatest improvements in individual parameters of the REVEAL risk score were in WHO FC (with 62.5% of patients improved, and where 40.3% of patients improved from WHO FC III to FC II), BNP or NT-proBNP (24.5% of patients improved), and 6-min walk test (23.9% of patients improved).

## Discussion

The REVEAL risk score calculator was originally developed to predict survival in patients with PAH [6]. Benza et al. [6] showed that low REVEAL risk stratum was associated with a 1-year survival rate of 95–100% while higher scores were associated with lower survival rates. The PRACMA study assessed the change in REVEAL risk score in PAH patients receiving macitentan for the first time in clinical practice. In our study, treatment with macitentan improved REVEAL risk score by risk stratum. The study population had an average to moderate high risk at baseline (mean risk score of 8.7), which diminished to low risk (mean risk score of 7.2) after a median time of treatment with macitentan of 11 months. There was a significant increase in the number of patients in the low risk stratum after at least 6-month follow-up period (20% more patients) and thus, an improvement in survival prognosis in these PAH patients was possible. On the other hand, around 23% less patients were in the highest risk strata after treatment with macitentan. Currently, other approaches to assess risk or risk stratification in PAH would include the use of risk variables according to the latest European Society of Cardiology (ESC)/ European Respiratory Society (ERS) guidelines for pulmonary hypertension [2], categorizing risk as low, intermediate or high with increasing estimated 1-year mortality. However, these guidelines were published after the PRACMA study had been started and thus, were not considered for risk analysis.

Moreover, treatment with macitentan significantly improved the REVEAL risk score in PAH patients with a mean decrease of 2 points in incident patients and a mean decrease of 1 point in prevalent patients. Improvements were maintained by treatment groups, either macitentan monotherapy, double combination with PDE5 inhibitor, or double or triple combination with prostacyclin subgroup. Overall, these findings are in accordance with previous data from the REVEAL cohort of Benza et al. [11] who found that incident patients were more likely to have decreased scores compared with prevalent patients (41% versus 28% of patients). These authors also reported 32% of patients with a decrease in the REVEAL risk score (58% in our study), 38% of patients with no change in the risk score (26% in our study), and 30% with an increase in the risk score (16% in our study) after 12 months of follow-up.

The REVEAL risk score has recently been applied to PAH patients treated with a guanylate cyclase stimulator [12], supporting its utility in predicting patient survival in a controlled population. Benza et al. [12] reported a significant change from baseline in REVEAL risk score (− 0.6 points) after 12 weeks of riociguat compared with placebo (− 0.1 points). By contrast, PRACMA was an observational study enrolling patients in real clinical practice and with longer follow-up.

By individual REVEAL component, treatment with macitentan significantly improved 6 out of 12 REVEAL components including WHO FC as well as the levels of BNP and NT-proBNP, and 6-min walk test results after at least six months of treatment, all of them determinants of PAH prognosis.

The previous phase III Seraphin study [10], which examined the efficacy and safety of macitentan, included a large number of PAH patients. Our population from several centres in Spain was representative of clinical practice for the treatment of PAH with macitentan. Overall, demographic data was consistent with that of the registries of PAH [13–15]. Comparison of demographic characteristics showed similar sex ratio but higher mean age in our population, 46 years in Seraphin study [10] versus 57 years in our study, probably due to the non-controlled design. Also, similar percentages of incident and prevalent patients among treatment-*naïve* patients were found in the Seraphin pivotal trial, 41 and 59%, respectively [16], although PAH treatment eras between enrolment in Seraphin and PRACMA studies were different. At 12-month follow-up of the REVEAL cohort [11], 35% of incident patients and 54% of prevalent patients were on combination therapy (consistent with 33 and 52% showed in our study), and 25% of incident patients and 34% of prevalent patients were receiving prostanoids. Although most of prevalent patients in our study were treated in combination therapy for PAH, there were 12 prevalent patients (35%) who reported macitentan as monotherapy. A switch from a previous endothelin receptor antagonist [17] could be one of the reasons. As expected, all patients on triple combination with a PDE5 inhibitor and a prostacyclin in clinical practice were prevalent.

Our study has several limitations. First, this was a retrospective study with data collection from medical records resulting in potential selection bias. Second, as idiopathic and heritable PAH were collected together, we were not able to differentiate familial PAH, which sums two points for the REVEAL risk score. This would not affect the change over time but the REVEAL score could be overestimated. Also, not all WHO group I PAH subgroups were included (e.g. PAH associated with portal hypertension). Third, we did not know for how long patients on combination therapy were receiving PDE5 inhibitor or prostacyclin nor if they were on stable doses prior to macitentan initiation. It is also important to note that concomitant diseases or medications were not recorded, and we were not able to assess their impact on the REVEAL risk score (for instance, diuretic treatment is known to be related to BNP levels [18]). Also, the PRACMA study did not evaluate the association of the risk score with survival. Finally, safety was not assessed (adverse events were not recorded in PRACMA study) because it was not a study objective.

Besides, current multifactorial assessment tools for measuring risk in PAH remain far from becoming mainstream tests in clinical practice. REVEAL risk score cannot be routinely applied to a high number of PAH patients. Recently, a modified risk assessment score of PAH, comprising four non-invasive variables, which could be more simply used in daily clinical setting than the REVEAL risk score has been published with promising results [19].

## Conclusion

In conclusion, in this study, treatment with macitentan improved the REVEAL risk strata and REVEAL risk score in incident and prevalent PAH patients, either in patients on monotherapy or on combination in clinical practice after at least six months of macitentan treatment. Further prospective studies are required to confirm these results and relate to survival prognosis in patients with PAH in clinical practice.

## Data Availability

The datasets used and/or analysed during the current study are available from the corresponding author on reasonable request.

## Abbreviations

BNP: Brain Natriuretic Peptide
DLCO: Diffusing Capacity of the Lungs for Carbon Monoxide
FC: Functional Class
mRAP: mean Right Atrial Pressure
NT-proBNP: N-terminal pro-Brain Natriuretic Peptide
PAH: Pulmonary Arterial Hypertension
PDE5: Phosphodiesterase Type 5
PVR: Pulmonary Vascular Resistance
REVEAL: Registry to Evaluate Early and Long-term PAH Disease Management
SBP: Systolic Blood Pressure
WHO: World Health Organization
WU: Wood Units
